# Monitoring the Evolution of Asynchrony between Mean Arterial Pressure and Mean Cerebral Blood Flow via Cross-Entropy Methods

**DOI:** 10.3390/e24010080

**Published:** 2022-01-02

**Authors:** Alberto Porta, Francesca Gelpi, Vlasta Bari, Beatrice Cairo, Beatrice De Maria, Cora May Panzetti, Noemi Cornara, Enrico Giuseppe Bertoldo, Valentina Fiolo, Edward Callus, Carlo De Vincentiis, Marianna Volpe, Raffaella Molfetta, Valeria Pistuddi, Marco Ranucci

**Affiliations:** 1 Department of Biomedical Sciences for Health, University of Milan, 20133 Milan, Italy; francesca.gelpi@grupposandonato.it (F.G.); beatrice.cairo@unimi.it (B.C.); edward.callus@grupposandonato.it (E.C.); 2 Department of Cardiothoracic, Vascular Anesthesia and Intensive Care, IRCCS Policlinico San Donato, 20097 San Donato Milanese, Italy; vlasta.bari@grupposandonato.it (V.B.); coramay.panzetti@gmail.com (C.M.P.); noemi.cornara@gmail.com (N.C.); valeria.pistuddi@grupposandonato.it (V.P.); marco.ranucci@grupposandonato.it (M.R.); 3 IRCCS Istituti Clinici Scientifici Maugeri, 20138 Milan, Italy; beatrice.demaria@icsmaugeri.it; 4 Clinical Psychology Service, IRCCS Policlinico San Donato, 20097 San Donato Milanese, Italy; enricogiuseppe.bertoldo@grupposandonato.it (E.G.B.); valentina.fiolo@grupposandonato.it (V.F.); 5 Department of Cardiac Surgery, IRCCS Policlinico San Donato, 20097 San Donato Milanese, Italy; carlo.devincentiis@grupposandonato.it; 6 Department of Cardiac Rehabilitation, IRCCS Policlinico San Donato, 20097 San Donato Milanese, Italy; marianna.volpe@grupposandonato.it (M.V.); raffaella.molfetta@grupposandonato.it (R.M.)

**Keywords:** state-space correspondence, cross-approximate entropy, cross-sample entropy, cerebrovascular control, cerebral autoregulation, beat-to-beat variability analysis, autonomic nervous system, active standing, surgical aortic valve replacement

## Abstract

Cerebrovascular control is carried out by multiple nonlinear mechanisms imposing a certain degree of coupling between mean arterial pressure (MAP) and mean cerebral blood flow (MCBF). We explored the ability of two nonlinear tools in the information domain, namely cross-approximate entropy (CApEn) and cross-sample entropy (CSampEn), to assess the degree of asynchrony between the spontaneous fluctuations of MAP and MCBF. CApEn and CSampEn were computed as a function of the translation time. The analysis was carried out in 23 subjects undergoing recordings at rest in supine position (REST) and during active standing (STAND), before and after surgical aortic valve replacement (SAVR). We found that at REST the degree of asynchrony raised, and the rate of increase in asynchrony with the translation time decreased after SAVR. These results are likely the consequence of the limited variability of MAP observed after surgery at REST, more than the consequence of a modified cerebrovascular control, given that the observed differences disappeared during STAND. CApEn and CSampEn can be utilized fruitfully in the context of the evaluation of cerebrovascular control via the noninvasive acquisition of the spontaneous MAP and MCBF variability.

## 1. Introduction

Given the importance of the brain and its high susceptibility to hypoxic states [1], the assessment of the dynamical relationship between mean cerebral perfusion pressure, approximated by the mean arterial pressure (MAP), and mean cerebral blood flow (MCBF), usually approximated by the MCBF velocity (MCBFV) assessed via the transcranial Doppler ultrasound device [2], is of paramount relevance. This evaluation is traditionally carried out by computing the degree of linear association between MAP and MCBFV as a function of the frequency via squared coherence function [3,4]. The difficulty of analyzing the degree of association between MAP and MCBFV is the consequence of the multiplicity of mechanisms operating along similar time scales that produce opposite effects on the strength of the MCBFV-MAP dynamical link [5]. Indeed, the pressure-to-flow relationship describes the MCBFV dynamics as a response to MAP fluctuations [6,7]. On the reverse time direction, the flow-to-pressure link, usually referred to as Cushing reflex [8], is responsible for increasing MAP via the activation of sympathetic circuits to avoid hypoperfusion associated, for example, with an increased intracranial pressure resulting from intracranial hemorrhage. However, a Cushing-like reflex might take place even in less dramatic conditions, with more limited variations of intracranial pressure [9,10,11]. The presence of the pressure-to-flow and flow-to-pressure links are responsible for the significant level of MCBFV-MAP association that allows the reliable estimation of the transfer function from MAP to MCBFV [12,13] and the computation of the strength of the causal link in both temporal directions [14,15,16,17,18]. However, an excessively high degree of MCBFV-MAP association might be unsafe in presence of a limited possibility of the brain to expand within the cranium [3]. As a consequence, the brain has developed powerful additional mechanisms of cerebrovascular control, that have been collectively termed as cerebral autoregulation (CA) [19,20]. CA counter-regulates vessel calibers to buffer MAP changes, with the aim at keeping unvaried MCBF. CA imposes a decoupling between MAP and MCBF variabilities, and the resulting limited degree of MCBFV-MAP association has been exploited to set specific ranges of perfusion pressure during cardiopulmonary bypass [21]. In addition to the multiplicity of mechanisms that have opposite influences on the strength of the MCBFV-MAP dynamical link, the listed regulatory mechanisms are highly nonlinear, given that the CA characteristic holds solely in a specific MAP range [1], MCBFV responses depend on the sign of MAP variations [22,23], MCBFV-MAP coupling varies with the breathing phase [24], and vagal and sympathetic controls are likely to non-additively interact each other in shaping CA [25,26].

Given the complexity of the cerebrovascular control, we propose to characterize the dynamical relationship between MAP and MCBFV via cross-entropy approaches [27]. We consider two tools, namely the cross-approximate entropy (CApEn) [28] and cross-sample entropy (CSampEn) [29]. CApEn and CSampEn are based on reconstructing the dynamics of the two series in two distinct state-spaces and on estimating probabilities to characterize the relationship between the two reconstructed state-spaces. These methods have the advantage of assessing the degree of asynchrony between the two series without assuming linearity.

Traditionally, CApEn and CSampEn fix the time translation *k* to 1 cardiac beat [28,29]. Indeed, when building the embedding space with dimension *m* the added component to the state-space with embedding dimension *m* − 1 was taken one-step-ahead in the future, with respect the most recent component of the pattern of dimension *m* − 1. Conversely, in this study, we are interested in quantifying the degree of asynchrony *k*-step-ahead into the future, and monitoring this marker with *k* under the hypothesis that the evolution of the marker with *k* might provide additional information about cerebrovascular control. In univariate analysis, the *k*-step-ahead prediction allows the characterization of the inherent nature of the dynamics, being the evolution of the normalized mean squared prediction error with *k* strongly linked to the largest positive Lyapunov exponent [30,31,32,33]. In the context of bivariate analysis, markers assessing *k*-step-ahead asynchrony based on cross-conditional entropy allowed us to unveil peculiar features of the baroreflex control during postural and pharmacological challenges [34,35].

The aim of the present study is to characterize cerebrovascular control via the degree of *k*-step-ahead asynchrony between MAP and MCBFV variability computed through CApEn and CSampEn. Data were acquired before (PRE) and after (POST) surgical aortic valve replacement (SAVR) [36].

## 2. Methods

### 2.1. Generalities for the Computation of CApEn and CSampEn

Given two systems X and Y, possibly interacting with each other, their mutual interactions are usually studied in real contexts by assessing the relationship between two stochastic process realizations *x* = {$x_{n}$, 1 ≤ *n* ≤ *N*} and *y* = {$y_{n}$, 1 ≤ *n* ≤ *N*} collected during experimental sessions. Defined as $y_{i}^{-}=\left[\begin{array}{ccc} y_{i-1} & \ldots & y_{i-m+1} \end{array}\right]$ the pattern formed by *m* − 1 past values of *y* and $y_{i-1+k}$ the *k*-step-ahead value of $y_{i}^{-}$ where *k* is the translation time, with 1 ≤ *k* ≤ *K*, where *K* is the maximum translation time, $y_{i-1+k}=y_{i-1+k}⊕y_{i}^{-}$ is the *m*-dimensional vector obtained by concatenating $y_{i-1+k}$ with $y_{i}^{-}$ with 1 ≤ *i* ≤ *N* − *m* − *k* + 2. Analogously, we define $x_{j}^{-}=\left[\begin{array}{ccc} x_{j-1} & \ldots & x_{j-m+1} \end{array}\right]$, $x_{j-1+k}$ and $x_{j-1+k}=x_{j-1+k}⊕x_{j}^{-}$ with 1 ≤ *j* ≤ *N* − *m* − *k* + 2. $y_{i}^{-}$ and $x_{j}^{-}$ can be interpreted as points of a (*m* − 1)-dimensional state-space built using the method of lagged coordinates, whereas $y_{i-1+k}$ and $x_{j-1+k}$ are points of a special *m*-dimensional space built non-uniformly [37,38] given that all the components of $y_{i}^{-}$ and $x_{j}^{-}$ are separated in time by *k* = 1, whereas the most recent sample of $y_{i-1+k}$ and $x_{j-1+k}$ are translated into the future by *k* when *k* > 1. We also define with $p\left(\|y_{i-1+k}-x_{j-1+k}\|\leq r\right)$ the probability that $y_{i-1+k}$ lies in the neighborhood of $x_{j-1+k}$ of size *r* and with $p\left(\|y_{i}^{-}-x_{j}^{-}\|\leq r\right)$ the probability that $y_{i}^{-}$ lies in the neighborhood of $x_{j}^{-}$ of size *r*, where *r* is the tolerance in the computation of the neighborhood and ‖·‖ is a metric to compute distance. In this study, the adopted metric is the maximum norm, namely the maximum of the absolute difference between corresponding scalar components [28,29].

### 2.2. CApEn

CApEn [28] is defined as the negative averaged logarithm of the ratio of the $p\left(\|y_{i-1+k}-x_{j-1+k}\|\leq r\right)$ to $p\left(\|y_{i}^{-}-x_{j}^{-}\|\leq r\right)$ as:

(1) $$\mathrm{CApEn}(m,r,N)=-\langle \log\left(\frac{p\left(\|y_{i-1+k}-x_{j-1+k}\|\leq r\right)}{p\left(\|y_{i}^{-}-x_{j}^{-}\|\leq r\right)}\right)\rangle$$

where 〈·〉 performs the average over all the reference vectors built over *x* and log is the natural logarithm. $p\left(\|y_{i-1+k}-x_{j-1+k}\|\leq r\right)$ and $p\left(\|y_{i}^{-}-x_{j}^{-}\|\leq r\right)$ are estimated as the fraction of $y_{i-1+k}$ in the neighborhood of the reference pattern $x_{j-1+k}$ and the fraction of $y_{i}^{-}$ in the neighborhood of the reference pattern $x_{j}^{-}$ within a tolerance *r*, respectively. The fractions are obtained by counting the number of $y_{i-1+k}$ closer than *r* to $x_{j-1+k}$ and the number of $y_{i}^{-}$ closer than *r* to $x_{j}^{-}$ and by dividing them by *N* − *m* − *k* + 2, respectively. The logarithm of $p\left(\|y_{i-1+k}-x_{j-1+k}\|\leq r\right)$ and of $p\left(\|y_{i}^{-}-x_{j}^{-}\|\leq r\right)$ was averaged over all the reference patterns built over *x* to obtain $\langle \log\left(p\left(y_{i-1+k}-x_{j-1+k}\leq r\right)\right)\rangle$ and $\langle \log\left(p\left(y_{i}^{-}-x_{j}^{-}\leq r\right)\right)\rangle$. In agreement with [29], we adopted the “bias 0” and “bias max” strategies to deal with the possibility that of $p\left(\|y_{i-1+k}-x_{j-1+k}\|\leq r\right)$ and/or $p\left(\|y_{i}^{-}-x_{j}^{-}\|\leq r\right)$ could be 0 due to the lack of $y_{i-1+k}$ and $y_{i}^{-}$ in the neighborhood of patterns $x_{j-1+k}$ and $x_{j}^{-},$ respectively. The “bias 0” strategy substituted the contemporaneous occurrence of $p\left(\|y_{i-1+k}-x_{j-1+k}\|\leq r\right)=0$ and $p\left(\|y_{i}^{-}-x_{j}^{-}\|\leq r\right)=0$ with $p\left(\|y_{i-1+k}-x_{j-1+k}\|\leq r\right)=1$ and $p\left(\|y_{i}^{-}-x_{j}^{-}\|\leq r\right)=1$. If only $p\left(\|y_{i-1+k}-x_{j-1+k}\|\leq r\right)=0$, $p\left(\|y_{i-1+k}-x_{j-1+k}\|\leq r\right)$ was set to ${\left(N-m-k+2\right)}^{-1}$ and $p\left(\|y_{i}^{-}-x_{j}^{-}\|\leq r\right)$ was left unaltered. The “bias max” strategy substituted $p\left(\|y_{i-1+k}-x_{j-1+k}\|\leq r\right)=0$ with $p\left(\|y_{i-1+k}-x_{j-1+k}\|\leq r\right)={\left(N-m-k+2\right)}^{-1}$ and $p\left(\|y_{i}^{-}-x_{j}^{-}\|\leq r\right)=0$ with $p\left(\|y_{i}^{-}-x_{j}^{-}\|\leq r\right)=1$. The CApEn is a measure of strength of association between *x* and *y*. Values of CApEn close to 0 indicate that patterns built over *x* and *y* that were close to *r* in the (*m* − 1)-dimensional space remained similar in the *m*-dimensional space. Conversely, high values of CApEn indicated a certain degree of asynchrony between *x* and *y*.

### 2.3. CSampEn

CSampEn [29] is defined as the negative logarithm of the ratio of the averaged $p\left(\|y_{i-1+k}-x_{j-1+k}\|\leq r\right)$ to the averaged $p\left(\|y_{i}^{-}-x_{j}^{-}\|\leq r\right)$ as:

(2) $$\mathrm{CSampEn}(m,r,N)=-\log\left(\frac{\langle p\left(\|y_{i-1+k}-x_{j-1+k}\|\leq r\right)\rangle }{\langle p\left(\|y_{i}^{-}-x_{j}^{-}\|\leq r\right)\rangle }\right),$$

where 〈·〉 performs the average over all the reference vectors built over *x*. At difference with CApEn $p\left(\|y_{i-1+k}-x_{j-1+k}\|\leq r\right)$ and $p\left(\|y_{i}^{-}-x_{j}^{-}\|\leq r\right)$ were averaged over all the reference patterns $x_{j-1+k}$ and $x_{j}^{-}$, respectively, to obtain $\langle p\left(\|y_{i-1+k}-x_{j-1+k}\|\leq r\right)\rangle$ and $\langle p\left(\|y_{i}^{-}-x_{j}^{-}\|\leq r\right)\rangle$ before carrying out the logarithm. The interpretation of CSampEn follows closely that of CApEn. The advantage is the smaller bias [29] given that it is unlikely that $\langle p\left(\|y_{i-1+k}-x_{j-1+k}\|\leq r\right)\rangle$ and/or $\langle p\left(\|y_{i}^{-}-x_{j}^{-}\|\leq r\right)\rangle$ were equal to 0. In the very unlikely case that $\langle p\left(\|y_{i-1+k}-x_{j-1+k}\|\leq r\right)\rangle$ was 0, it was substituted with ${\left(N-m-k+2\right)}^{-2}$. In the equally unlikely situation that both $\langle p\left(\|y_{i-1+k}-x_{j-1+k}\|\leq r\right)\rangle$ and $\langle p\left(\|y_{i}^{-}-x_{j}^{-}\|\leq r\right)\rangle$ were 0, the ratio of $\langle p\left(\|y_{i-1+k}-x_{j-1+k}\|\leq r\right)\rangle$ to $\langle p\left(\|y_{i}^{-}-x_{j}^{-}\|\leq r\right)\rangle$ was to set to ${\left(N-m-k+2\right)}^{-2}$.

## 3. Experimental Protocol and Data Analysis

### 3.1. Experimental Protocol

The study was in keeping with the Declaration of Helsinki. The study was approved by the ethical review board of the San Raffaele Hospital, Milan, Italy (approval number: 68/int/2018; approval date: 5 April 2018) and authorized by the Policlinico San Donato, San Donato Milanese, Milan, Italy (authorization date: 13 April 2018). Written, signed and informed consent was obtained from all subjects.

We enrolled 30 patients (age: 66 ± 10 yrs, 23 males) undergoing SAVR at the IRCCS Policlinico San Donato, San Donato Milanese, Milan, Italy. Demographic and clinical data of the SAVR group are reported in Table 1. They did not feature either atrial fibrillation, overt autonomic nervous system pathologies or cerebrovascular diseases. We acquired electrocardiogram (ECG) from lead II (BioAmp FE132, ADInstruments, Bella Vista New South Wales, Australia), non-invasive finger arterial pressure (AP) by volume-clamp photoplethysmography (CNAP Monitor 500, CNSystems, Graz, Austria), and pulsatile cerebral blood-flow velocity (CBFV) via transcranial Doppler device (Multi-Dop X, DWL, San Juan Capistrano, CA, USA) from the left or right middle cerebral artery. Signals were sampled at 400 Hz through a commercial acquisition system (Power Lab, ADInstruments, Bella Vista New South Wales, Australia). Signals were recorded in PRE, i.e., 1 day before SAVR, and in POST, i.e., within 7 days after SAVR, at rest in supine position (REST) and during active standing (STAND). Experimental sessions lasted 10 min with REST acquired always before STAND. Seven patients were excluded due to poor quality of CBFV, as checked during the first session of the protocol (i.e., at REST in PRE). PRE analyses could be carried out in 23 subjects at REST, and in 20 individuals during STAND subjects, whereas POST data was processed for 15 and 13 patients, respectively. The decreasing number of subjects in POST compared with PRE is explained by the post-surgery physical and psychological debilitation of some patients. The difficulty in locating either the left or right middle cerebral artery in the different experimental sessions further limited the final figures. We checked that the groups examined in the PRE and POST conditions exhibited the same basic characteristics as the population reported in the Table 1 (e.g., demographic data). This consideration held for the comparison between individuals at REST and during STAND. Pharmacological treatment that might interfere with the autonomic control was preserved in POST, unless specific situations suggested the administration of additional medications (e.g., beta-blockers in case of associated coronary artery bypass graft surgery). No significant differences were found across groups in relation to pharmacological treatment that might interfere with the autonomic nervous system activity.

### 3.2. Extraction of Beat-to-Beat Variability Series

The ECG was exploited to facilitate the detection of diastolic fiducial points on AP. After detecting the R-wave peaks from the ECG using a threshold applied to the first derivative, the *n*th systole and diastole were located, respectively, as the timing of the AP maximum was observed within the *n*th cardiac cycle, and the timing of the AP minimum following the *n*th systole. The AP at the systolic and diastolic points were taken as systolic AP (SAP) and diastolic AP (DAP). The length of the *n*th heart period (HP) was also derived as the time interval between the *n*th and (*n* + 1)th R-wave peaks. The *n*th MAP was obtained as the integral of AP between the (*n* − 1)th and *n*th diastoles, and by dividing the result by the corresponding interdiastolic time interval. The *n*th MCBFV was computed similarly over the CBFV signal using the minima detected over the CBFV signal in the proximity of diastoles. The HP, SAP, DAP, MAP and MCBFV series were manually checked and corrected in case of missing beats or misdetections. Effects of ectopic beats or isolated arrhythmic events were mitigated via linear interpolation. Synchronous sequences lasting 256 consecutive beats were randomly selected within the whole recordings.

### 3.3. Computation of Variability Markers

In time domain we computed the means, indicated as μ_HP_, μ_SAP_, μ_DAP_, μ_MAP_ and μ_MCBFV_, and the variances, denoted with σ^2^_HP_, σ^2^_SAP_, σ^2^_DAP_, σ^2^_MAP_ and σ^2^_MCBFV_. Means and variances were expressed, respectively, in ms, mmHg, mmHg, mmHg and cm·s^−1^ and ms^2^, mmHg^2^, mmHg^2^, mmHg^2^ and cm^2^·s^−2^. After computing the mean, the series were linearly detrended before computing variance and asynchrony markers. 

CApEn and CSampEn were computed over normalized series obtained by subtracting the mean to each value and dividing the result by the standard deviation *σ*. According to the standard setting [28,29,39] we assigned *m* = 3 and *r* = 0.2 × *σ*. CApEn and CSampEn were computed as a function of *k* with 1 ≤ *k* ≤ *K* and *K* = 8. CApEn and CSampEn at *k* = 1 were denoted as CApEn_*k*=1_ and CSampEn_*k*=1_, respectively. Linear regression analysis of CApEn and CSampEn on *k* was performed on an individual basis. The slopes of the linear regression were computed, and these markers were denoted as CApEn_slope_ and CSampEn_slope_.

### 3.4. Statistical Analysis

Two-way analysis of variance (Holm–Sidak test for multiple comparisons) was applied to CApEn and CSampEn markers to assess the effect of translation time versus *k* = 1 within the same period of analysis (i.e., PRE or POST) and the effect of the surgery within the same translation time (from 1 to 8). Two-way analysis of variance (Holm–Sidak test for multiple comparisons) was applied to CApEn_*k*=1_, CSampEn_*k*=1_, CApEn_slope_ and CSampEn_slope_ to detect the effect of cardiac surgery within the same experimental condition (i.e., REST or STAND) and the response to the postural challenge within the same period of analysis (i.e., PRE or POST). Statistical analysis was carried out using a commercial statistical program (Sigmaplot, v.14.0, Systat Software, Inc., Chicago, IL, USA). A *p* < 0.05 was always considered statistically significant.

## 4. Results

Table 2 summaries the time domain markers derived from HP, SAP, DAP, MAP and MCBFV. STAND reduced µ_HP_, but this effect was visible only in PRE. STAND increased µ_DAP_ exclusively in POST. The depression of cardiovascular autonomic control was stressed by the decrease in σ^2^_HP_ and σ^2^_DAP_ in POST at REST. The expected increase in σ^2^_SAP_ and of σ^2^_DAP_, and the expected decline in σ^2^_HP_ with STAND was not detected in either PRE or POST. None of the time domain markers usually evaluated to typify CA, namely µ_MAP_, σ^2^_MAP_, µ_MCBFV_ and σ^2^_MCBFV_ varied with orthostatic challenge and/or period of analysis.

In the following we reported results of CApEn derived exclusively using “bias 0” strategy. Indeed, findings relevant to the use of “bias max” strategy exhibited similar differences between periods of analysis and/or experimental conditions.

**Figure 1:**
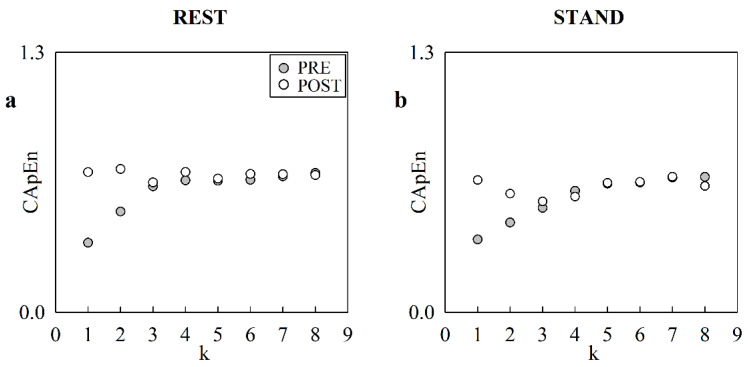
The scatter plots represent the course of CApEn as a function of the translation time *k* in a representative subject at REST (**a**), and during STAND (**b**). The solid circles are relevant to PRE and the open circles refer to POST.

The scatter plots in Figure 1 show the course of CApEn as a function of the translation time *k* at REST (Figure 1a) and during STAND (Figure 1b) in a representative subject. The trend of CApEn in PRE is given as solid circles, whereas in POST it is given as open circles. CApEn starts from lower values and the rate of the CApEn increase was faster in PRE than POST, both at REST and during STAND.

**Figure 2:**
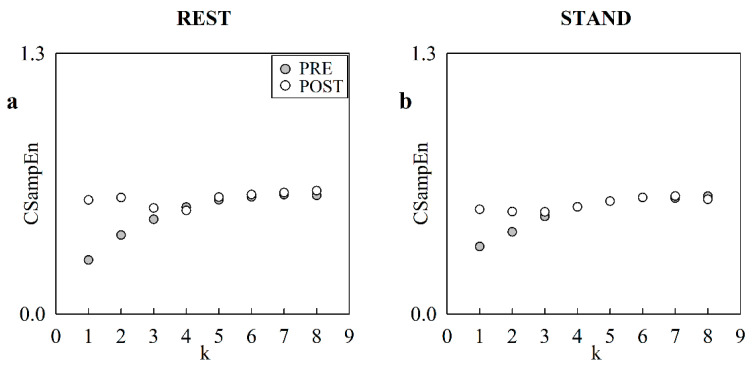
The scatter plots represent the course of CSampEn as a function of the translation time *k* in a representative subject at REST (**a**), and during STAND (**b**). The solid circles are relevant to PRE and the open circles refer to POST.

Figure 2 has the same structure as Figure 1, but it shows the course of CSampEn as a function of the translation time *k* at REST (Figure 2a) and during STAND (Figure 2b) in the same representative subject. The evolutions of CSampEn with *k* at REST are similar to those reported in Figure 1a, whereas during STAND the trends of CSampEn in PRE and POST are more similar.

**Figure 3:**
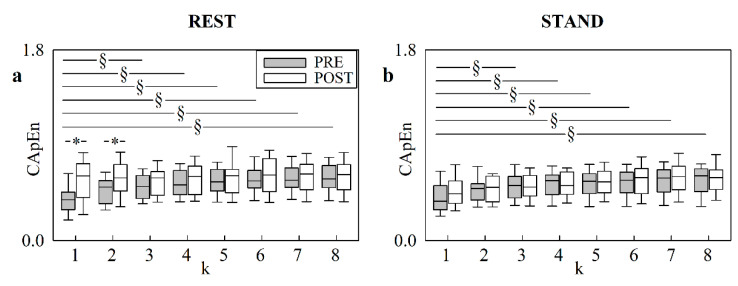
The box-and-whisker plots shows show CapEn at REST (**a**), and during STAND (**b**), as a function of the translation time *k* (i.e., from 1 to 8). The markers are given in PRE (grey boxes) and POST (white boxes). The symbol § indicates a significant difference versus *k* = 1 with *p* < 0.05. The symbol * indicates a significant difference versus PRE with *p* < 0.05.

The vertical box-and-whisker plots of Figure 3 show CApEn as a function of the translation time *k* at REST (Figure 3a) and during STAND (Figure 3b). The markers of asynchrony were reported in PRE (grey boxes) and in POST (white boxes). The height of the box represents the distance between the first and third quartiles, with the median marked as a line, and the whiskers show the 5th and 95th percentiles. In PRE, CApEn increased with *k*: the raise compared to *k* = 1 was significant for k ≥ 3 both at REST and during STAND. In POST, CApEn remained stable with *k* and the finding held both at REST and during STAND. At REST, CApEn was significantly larger in POST than in PRE solely at *k* = 1 and *k* = 2, whereas during STAND no PRE–POST differences were visible, regardless of the values of *k*.

**Figure 4:**
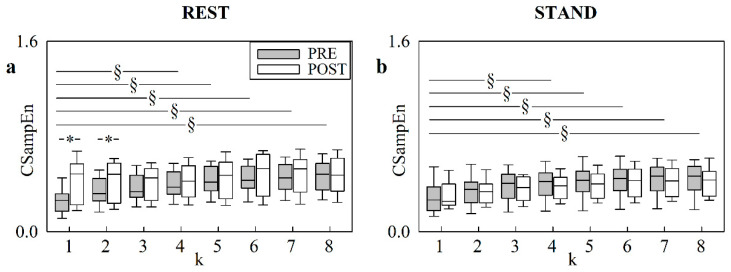
The box-and-whisker plots show CSampEn at REST (**a**), and during STAND (**b**), as a function of the translation time *k* (i.e., from 1 to 8). The markers are given in PRE (grey boxes) and POST (white boxes). The symbol § indicates a significant difference versus *k* = 1 with *p* < 0.05. The symbol * indicates a significant difference versus PRE with *p* < 0.05.

Figure 4 has the same structure as Figure 3, but it shows the course of CSampEn with *k* at REST (Figure 4a) and during STAND (Figure 4b). Results were similar to those reported for CApEn. The exclusive difference of Figure 4 compared with Figure 3 is that the increase in CSampEn compared with *k* = 1, detectable in PRE, was evident for *k* ≥ 4. 

**Figure 5:**
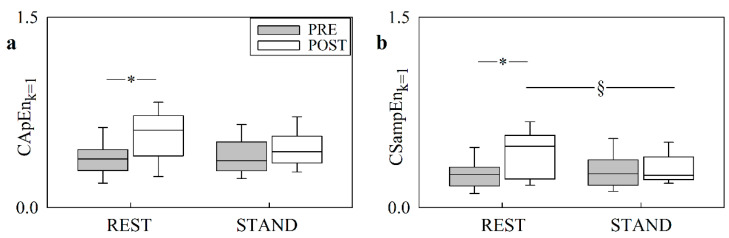
The box-and-whisker plots show CApEn_*k*=1_ (**a**), and CSampEn_*k*=1_ (**b**), as a function of the experimental condition (i.e., REST and STAND). The markers are given in PRE (grey boxes) and POST (white boxes). The symbol § indicates a significant difference versus REST with *p* < 0.05. The symbol * indicates a significant difference versus PRE with *p* < 0.05.

The vertical box-and-whisker plots of Figure 5 show CApEn_*k*=1_ (Figure 5a) and CSampEn_*k*=1_ (Figure 5b) as a function of the experimental condition (i.e., REST and STAND). The markers of asynchrony were reported in PRE (grey boxes) and in POST (white boxes). The height of the box represents the distance between the first and third quartiles, with the median marked as a line, and the whiskers show the 5th and 95th percentiles. CApEn_*k*=1_ increased in POST compared with PRE at REST, whereas the effect of cardiac surgery was not evident during STAND. The same conclusion held for CSampEn_*k*=1_. In PRE, the postural challenge did not induce any modification of either CApEn_*k*=1_ or CSampEn_*k*=1_. Conversely, in POST we observed a tendency towards a decrease in markers of asynchrony during STAND, compared with REST, and this tendency became significant in the case of CSampEn_*k*=1_.

**Figure 6:**
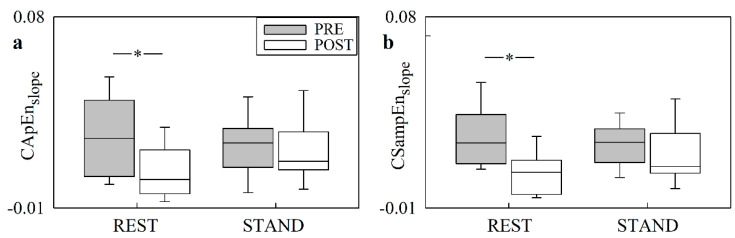
The box-and-whisker plots show CApEn_slope_ (**a**), and CSampEn_slope_ (**b**), as a function of the experimental condition (i.e., REST and STAND). The markers are given in PRE (grey boxes) and POST (white boxes). The symbol * indicates a significant difference versus PRE with *p* < 0.05.

Figure 6 has the same structure as Figure 5, but it shows CApEn_slope_ (Figure 6a) and CSampEn_slope_ (Figure 6b). Similar to Figure 5, the effect of cardiac surgery was significant only at REST over both asynchrony markers. STAND did not affect either CApEn_slope_ or CSampEn_slope_, regardless of the period of analysis.

## 5. Discussion

The main findings of this study can be summarized as follows: (i) CApEn and CSampEn allow the assessment of the asynchrony between two time series as a function of the translation time; (ii) CApEn and CSampEn provide similar conclusions about the effect of postural challenge and cardiac surgery; (iii) none of the time domain markers characterizing MCBFV-MAP regulation detect the impact of either cardiac surgery or postural challenge; (iv) the impact of cardiac surgery on asynchrony markers is evident at REST but irrelevant during STAND; (v) postural challenge has a limited impact on asynchrony markers visible only over CSampEn_*k*=1_ in POST.

### 5.1. CApEn and CSampEn Allow the Assessment of the MCBFV-MAP Asynchrony as a Function of the Translation Time

In the study of cerebrovascular dynamical interactions, a reliable quantification of the degree of MCBFV-MAP association is fundamental. Indeed, the strength of MCBFV-MAP coupling is the balance between CA mechanisms that should induce a certain degree of decoupling between MAP and MCBFV variability series, given that CA aims at limiting the variability of MCBFV against MAP variations [3,4,19,20,21], and the high level of association imposed by the pressure-to-flow [6,7] and flow-to-pressure [8,9,10,11] causal pathways. This study originally applied CApEn and CSampEn to quantify the degree of asynchrony between the MAP and MCBFV variability series, and monitor the level of MCBFV-MAP asynchrony as a function of the translation time *k*. CApEn and CSampEn built separate patterns of dimension *m* − 1 over both MAP and MCBFV variability series, and checked whether these patterns remained close after the enlargement of both patterns with an additional component *k*-step-ahead into the future. This feature means that CApEn and CSampEn belong to the class of state-space correspondence methods that propose to reconstruct the dynamical behaviors of two series in two separate embedding spaces, and search for the possible relationship linking the reconstructed geometrical entities [40,41,42]. The advantage of model-free approaches based on state-space correspondence lies in the possibility of the searching association under very broad hypotheses [40,41,42]. Therefore, this approach might be well suited in the context of assessment of cerebrovascular control and CA, given the nonlinear characteristics of the link between MAP and MCBFV [1,22,23,24,25,26]. Moreover, the proposed approach has the advantage of assessing asynchrony as a function of *k*. Asynchrony between two stochastic processes is expected to change with the dynamical characteristic of the input–output relationship between them. Therefore, characterization of the evolution of asynchrony with *k* might provide new indexes describing the MCBFV-MAP relationship. In general, in stochastic systems, the difficulty in predicting the output from the input increases with prediction time, and this characteristic leads to a level of asynchrony between the input and the output increasing with *k* [43]. It has been demonstrated that, in the presence of a full decoupling between the input and the output, asynchrony assessed via cross-conditional entropy did not vary with *k* [34,35]. This finding holds even when asynchrony is evaluated via mutual predictability [40]. Moreover, it was found that the presence of a nonlinear relationship between the input and the output might influence the value of asynchrony and the rate of increase in asynchrony with *k* [35]. As a matter of fact, a cross-condition entropy approach capable of describing nonlinearities detected a smaller level of asynchrony and a higher rate of rise of asynchrony with *k* than those found over surrogate data built from the original ones by preserving only linear aspects of the dynamics and their interactions such as cross-correlation, power spectra and distributions [35].

### 5.2. CApEn and CSampEn Provide Similar Conclusions about the Effect of Postural Challenge and Cardiac Surgery

Both CApEn and CSampEn measure the degree of asynchrony between two time series [28,29]. Despite being defined to measure the same quality of the dynamical interactions between two time series, there are a couple of reasons that might lead CApEn and CSampEn to different conclusions. First, CApEn needs the application of correction schemes [29] to enlarge its original definition [28], that is otherwise very limited due to the highly likely occurrence of the log-of-zero situation [29] and the untrustworthiness of procedures designed to increase the number of matches [44]. Second, the CApEn is a directional marker, whereas CSampEn is not. Indeed, even though $p\left(\|y_{i-1+k}-x_{j-1+k}\|\leq r\right)$ and $p\left(\|y_{i}^{-}-x_{j}^{-}\|\leq r\right)$ are directional markers (i.e., reversing the role of *x* and *y* modified probabilities), $\langle p\left(\|y_{i-1+k}-x_{j-1+k}\|\leq r\right)\rangle$ and $\langle p\left(\|y_{i}^{-}-x_{j}^{-}\|\leq r\right)\rangle$ are direction-independent, given that they are equivalent to the probability of finding pairs of vectors built over *x* and *y*, at a distance closer than *r* in the *m*-dimensional and (*m* − 1)-dimensional embedding spaces, respectively, namely a feature that evidently does not depend on which series is taken as *x* or *y*. Conversely, the directionality of CApEn is the consequence of the application of the logarithm to $p\left(\|y_{i-1+k}-x_{j-1+k}\|\leq r\right)$ and $p\left(\|y_{i}^{-}-x_{j}^{-}\|\leq r\right)$ before averaging, and to its nonlinear characteristic. Contrary to the expectations, results derived from CApEn and CSampEn were similar. Indeed, both CApEn and CSampEn markers were able to detect the impact of cardiac surgery at REST, but not during STAND, and trends with posture modification were similar. Therefore, we can conclude that in the context of evaluating cerebrovascular control in SAVR population there is no reason to privilege either approach.

### 5.3. Impact of CApEn and CSampEn on the Assessment of the Cerebrovascular Control in SAVR Patients

This study confirms previous results on cardiac, vascular, and cerebrovascular controls in the SAVR population [36,45,46,47]. Time domain markers suggest that cardiac and vascular regulations are depressed after SAVR surgery, as indicated by the decline of σ^2^_HP_ and σ^2^_DAP_ and by the irrelevant modifications of σ^2^_HP_, σ^2^_SAP_ and σ^2^_DAP_ in response to STAND. The missing impact of STAND over σ^2^_HP_, σ^2^_SAP_ and σ^2^_DAP_ in PRE indicates that cardiovascular control is impaired already before SAVR surgery [36]. Time domain markers confirm that cerebrovascular control and CA were not affected by the postural challenge and cardiac surgery given that, in the presence of stable values of σ^2^_MAP_ with postural and surgical stressors, σ^2^_MCBFV_ remained unvaried [36].

Both CApEn and CSampEn were able to detect at REST the greater MCBFV-MAP asynchrony in POST, compared with PRE. The same tendency was found via squared coherence computed in the frequency bands below the respiratory one [36], but in the present study the change is significant. This finding might be taken as an indication of the postoperative improvement of CA, given that the aim of CA is to limit variability of MCBFV against MAP variations [3,4,19,20,21]. Indeed, situations of impaired CA, such as those induced by aneurysmal subarachnoid hemorrhage, and by pharmacological autonomic blockades increased the squared coherence function between 0.04 and 0.08 Hz [3,25,26]. However, this interpretation holds in the presence of significant MAP variations. Since we observed a post-surgery sympathetic depression in our population resulting in a tendency toward lower amplitudes of MAP oscillations, this input might be insufficient to drive MCBFV changes. The unvaried modification of MCBFV-MAP asynchrony in POST compared to PRE during STAND corroborates the conclusion that cardiac surgery did not modify CA [36]. Indeed, during STAND the amplitude of the MAP oscillations tended to increase, compared with REST in our population [36], and this increase might be able to drive MCBFV oscillations, thus revealing that the degree of association between MAP and MCBFV was not changed after cardiac surgery.

Both CApEn and CSampEn detected a smaller rate of increase in MCBFV-MAP asynchrony with translation time *k* at REST in POST compared with PRE. The reduced increase in asynchrony with *k* between MAP and MCBFV variability might indicate a more deterministic relation between MCBFV and MAP, or a more limited ability to reach the condition of MCBFV-MAP uncoupling in POST than in PRE. Given that the MCBFV-MAP link is the result of the integrate action of multiple mechanisms comprising chemoreflex, neuronal metabolism, neurovascular coupling, CA and autonomic control [5], the tendency toward a more deterministic MCBFV-MAP relation might indicate a postoperative loss of cerebrovascular complexity and, consequently, an impairment of cerebrovascular regulation. Given that the goal of CA is to limit the variability of MCBFV despite changes in MAP [3,4,19,20,21], the tendency towards a reduced rate of increase in the MCBFV-MAP decoupling with *k* might suggest an impairment of cerebrovascular control. However, again the insufficient perturbing action of MAP might be responsible for this finding. This conclusion is corroborated by the missed postoperative variations of CApEn_slope_ and CSampEn_slope_ during STAND, when MAP changes tended to be more important. 

The effect of STAND on asynchrony markers is limited, and this conclusion is in agreement with several studies stressing the negligible impact of the postural challenge over cerebrovascular regulation [48,49,50]. Indeed, solely CSampEn_*k*=1_ indicated an effect of STAND, and this influence was detected solely in POST. However, since a similar tendency was suggested by CApEn_*k*=1_ as well, and given that it was evident only in POST, future studies carried out should investigate more deeply the effect of posture change in this population by considering a larger number of subjects.

### 5.4. Limitations of the Study and Future Developments

A possible limitation of the study is the missed random allocation of the subject in each condition. Indeed, the allocation of a subject in each group is exclusively based on the quality of the recording of AP and CBFV. This criterion might have biased the study toward a special subset of our original group, despite the preservation of the general characteristic of the overall population. Another possible limitation of the study is linked to the dependence of the autonomic response on the number of postoperative days [51]. In the present study the timing of POST varies from 4 to 7 days. This peculiar setting is likely to have increased the variance of the markers, but it is unlikely to have biased the study towards a specific conclusion given that the autonomic control is expected to remain influenced by surgery for some days [51]. One possible confounding factor is the significant fraction of subjects under beta-adrenergic blockade in our population. However, the impact of beta-blockers on CA is more controversial [52,53] than that of alpha-blockers [25]. The impact of beta-blocker therapy on the PRE–POST and/or REST-STAND comparisons is expected to be limited, given that the fraction of subjects under beta-blocker therapy is similar in all conditions. Future studies should test this approach in subjects who developed stroke during SAVR surgery, to test whether the method could indicate a CA impairment.

## 6. Conclusions

The link between MAP and MCBFV spontaneous fluctuations resulting from the action of cerebrovascular control mechanisms was explored via cross-entropy approaches. Cross-entropy metrics have the possibility to interpret possible nonlinear interactions between the two series and monitor the evolution of the degree of the MCBFV-MAP asynchrony with the translation time. The approach was found to be useful to typify cerebrovascular regulation. More specifically, since the effect of cardiac surgery on cross-entropy markers observed at REST disappeared during STAND, we conclude that cardiac surgery did not alter the state of the cerebrovascular regulation in SAVR population. 

## Figures and Tables

**Table 1:** Clinical and demographic markers of SAVR patients.

Index	SAVR (*n* = 30)
Age [yrs]	66 ± 10
Gender [male]	23 (77)
Weight [kg]	78.5 ± 16.2
BMI [kg·m^−2^]	27.2 ± 4.8
Congestive heart failure	1 (3)
Recent myocardial infarction	0 (0)
Previous cerebrovascular events	1 (3)
LVEF [%]	58.6 ± 10.4
Diabetes	3 (10)
COPD	3 (10)
Serum creatinine [mg·dL^−1^]	0.99 ± 0.34
Hypertension	14 (47)
HCT [%]	41.7 ± 4.6
ACE inhibitors	9 (32)
Beta-blockers	16 (57)
Diuretics	8 (29)
Calcium antagonists	1 (3)
Antiarrhythmic drugs	0 (0)
Combined intervention	15 (54)
EuroSCORE II	2.3 ± 2.3
CPB time [minutes]	94.1 ± 35.3
Nadir temperature on CPB [°C]	33.5 ± 1.3
Catecholamine administration	2 (7)
Mechanical ventilation time [hours]	10.6 ± 5.4
ICU stay [days]	1.5 ± 0.9
Hospital stay [days]	7.6 ± 2.9
Postoperative atrial fibrillation	11 (37)
Postoperative arrhythmias	2 (7)
Postoperative low cardiac output syndrome	2 (7)
Postoperative stroke	0 (0)
Postoperative acute kidney injury	0 (0)

SAVR = surgical aortic valve replacement; BMI = body mass index; LVEF = left ventricular ejection fraction; COPD = chronic obstructive pulmonary disease; HCT = hematocrit; ACE = angiotensin converting enzyme; EuroSCORE = European System for Cardiac Operative Risk Evaluation; CPB = cardiopulmonary bypass; ICU = intensive care unit. Continuous data are presented as mean ± standard deviation and categorical data as number (percentage).

**Table 2:** Time domain markers.

Index	PRE		POST	
	REST	STAND	REST	STAND
μ_HP_ [ms]	975 ± 132	865 ± 119 §	780 ± 122 *	712 ± 129 *
σ^2^_HP_ [ms^2^]	917 ± 835	748 ± 542	274 ± 535 *	368 ± 641
μ_SAP_ [mmHg]	140 ± 18	134 ± 20	134 ± 16	137 ± 22
σ^2^_SAP_ [mmHg^2^]	26 ± 21	43 ± 43	25 ± 17	32 ± 16
μ_DAP_ [mmHg]	70 ± 13	75 ± 18	69 ± 18	86 ± 24 §
σ^2^_DAP_ [mmHg^2^]	14 ± 13	14 ± 13	6 ± 4 *	11 ± 7
μ_MAP_ [mmHg]	97 ± 13	96 ± 17	90 ± 19	101 ± 23
σ^2^_MAP_ [mmHg^2^]	18 ± 13	24 ± 18	15 ± 9	17 ± 8
μ_MCBFV_ [cm·s^−1^]	57 ± 26	49 ± 23	62 ± 39	58 ± 34
σ^2^_MCBFV_ [cm^2^·s^−2^]	25 ± 33	22 ± 21	21 ± 20	22 ± 23

HP = heart period; SAP = systolic arterial pressure; DAP = diastolic arterial pressure; MAP = mean arterial pressure; MCBFV = mean cerebral blood flow velocity; µ_HP_ = HP mean; σ^2^_HP_ = HP variance; µ_SAP_ = SAP mean; σ^2^_SAP_ = SAP variance; µ_DAP_ = DAP mean; σ^2^_DAP_ = DAP variance; µ_MAP_ = MAP mean; σ^2^_MAP_ = MAP variance; µ_MCBFV_ = MCBFV mean; σ^2^_MCBFV_ = MCBFV variance; SAVR = surgical aortic valve replacement; PRE = 1 day before SAVR surgery; POST = within 7 days after SAVR surgery. REST = at rest in supine position; STAND = during standing. The symbol * indicates a significant difference versus PRE with *p* < 0.05. The symbol § indicates a significant difference versus REST with *p* < 0.05.

## Data Availability

The data presented in this study are available on request from the corresponding author (i.e., A.P.) upon permission of the IRCCS Policlinico San Donato. The data are not publicly available because they contain information that could compromise the privacy of research participants.
